# Scalable manufacturing of gene-modified human mesenchymal stromal cells with microcarriers in spinner flasks

**DOI:** 10.1007/s00253-023-12634-w

**Published:** 2023-07-20

**Authors:** Pedro Silva Couto, Dale J. Stibbs, Marco C. Rotondi, Yasuhiro Takeuchi, Qasim A. Rafiq

**Affiliations:** 1 Department of Biochemical Engineering, Advanced Centre for Biochemical Engineering, University College London, Gower Street, London, WC1E 6BT UK; 2 Division of Infection and Immunity, University College London, Gower Street, London, WC1E 6BT UK; 3 Biotherapeutics and Advanced Therapies, Scientific Research and Innovation, Medicines, and Healthcare Products Regulatory Agency, South Mimms, EN6 3QG UK

**Keywords:** Engineering, Mesenchymal, Manufacturing, Lentiviral vector, Microcarrier, Gene-modified

## Abstract

**Abstract:**

Due to their immunomodulatory properties and *in vitro* differentiation ability, human mesenchymal stromal cells (hMSCs) have been investigated in more than 1000 clinical trials over the last decade. Multiple studies that have explored the development of gene-modified hMSC-based products are now reaching early stages of clinical trial programmes. From an engineering perspective, the challenge lies in developing manufacturing methods capable of producing sufficient doses of *ex vivo* gene-modified hMSCs for clinical applications. This work demonstrates, for the first time, a scalable manufacturing process using a microcarrier-bioreactor system for the expansion of gene-modified hMSCs. Upon isolation, umbilical cord tissue mesenchymal stromal cells (UCT-hMSCs) were transduced using a lentiviral vector (LV) with green fluorescent protein (GFP) or vascular endothelial growth factor (VEGF) transgenes. The cells were then seeded in 100 mL spinner flasks using Spherecol microcarriers and expanded for seven days. After six days in culture, both non-transduced and transduced cell populations attained comparable maximum cell concentrations (≈1.8 × 10^5^ cell/mL). Analysis of the culture supernatant identified that glucose was fully depleted after day five across the cell populations. Lactate concentrations observed throughout the culture reached a maximum of 7.5 mM on day seven. Immunophenotype analysis revealed that the transduction followed by an expansion step was not responsible for the downregulation of the cell surface receptors used to identify hMSCs. The levels of CD73, CD90, and CD105 expressing cells were above 90% for the non-transduced and transduced cells. In addition, the expression of negative markers (CD11b, CD19, CD34, CD45, and HLA-DR) was also shown to be below 5%, which is aligned with the criteria established for hMSCs by the International Society for Cell and Gene Therapy (ISCT). This work provides a foundation for the scalable manufacturing of gene-modified hMSCs which will overcome a significant translational and commercial bottleneck.

**Key points:**

• *hMSCs were successfully transduced by lentiviral vectors carrying two different transgenes: GFP and VEGF*

• *Transduced hMSCs were successfully expanded on microcarriers using spinner flasks during a period of 7 days*

• *The genetic modification step did not cause any detrimental impact on the hMSC immunophenotype characteristics*

**Supplementary Information:**

The online version contains supplementary material available at 10.1007/s00253-023-12634-w.

## Introduction

Cell and gene therapy (CGT) represents a novel and potentially transformative therapeutic modality. However, these CGT products have become increasingly complex to manufacture. This is in part due to the range of cellular modalities investigated (Heathman et al. 2015; Silva Couto et al. 2017; Couto et al. 2019), as well as the need for genetically engineering the cells to integrate the gene of interest, responsible for eliciting the desired clinical effect (Wang and Rivière 2016; Costariol et al. 2020). Other novel approaches using products of cells (e.g., extracellular vesicles) have also been explored in recent clinical trials (Shi et al. 2021; Gupta et al. 2021; Fathi-Kazerooni et al. 2022). The field has seen the recent successes of chimeric antigen receptor T cell immunotherapies (CAR-T) receiving approval to target haematological malignancies, such as acute lymphoblastic leukaemia (ALL), B-cell lymphoma, follicular lymphoma, mantle cell lymphoma, and multiple myeloma (Mullard 2021; Sengsayadeth et al. 2022; Strati 2022).

Human mesenchymal stromal cells (hMSCs) are a promising candidate amongst the non-blood related cell types employed in CGT applications. Given their *in vitro* differentiation ability and immunomodulatory properties (Tamama et al. 2008; Da Silva Meirelles et al. 2008; Caplan and Correa 2011; Munir et al. 2017), hMSCs have been explored in several clinical trials during the last decade (Silva Couto et al. 2017; Kabat et al. 2020). These biological characteristics place hMSCs in a unique position as a candidate for cell therapy and tissue engineering applications. Although these cells can be isolated from both adult (adipose tissue (AT), bone marrow (BM)) and perinatal tissues (umbilical cord tissue (UCT) and placenta for example), multiple studies have reported biological differences across sources of hMSCs (Barlow et al. 2008; Ikegame et al. 2011; Jin et al. 2013; Mattar and Bieback 2015; Du et al. 2016).

The lack of evidence for *in vivo *engraftment and differentiation (Grinnemo et al. 2006; Von Bahr et al. 2012; Gomez-Salazar et al. 2020) together with the observation that hMSCs produce a wide range of immunomodulatory molecules has suggested that their mechanism of action (MoA) is attributed to the cells’ paracrine signalling capability (Caplan and Correa 2011; Mattar and Bieback 2015; Wang et al. 2018). However, it remains unclear whether hMSC immunomodulatory properties are the result of cell to cell interactions or exclusively driven by the cell secretome (Nasef et al. 2006; Zanotti et al. 2013; Mallis et al. 2021).

From a clinical perspective, hMSC-based products have already been approved worldwide (Silva Couto et al. 2020a) targeting conditions such as acute myocardial infarction, graft *versus* host disease, and degenerative arthritis, amongst others. Although most clinical trials use hMSCs as a cell therapy product, the application of this cell type for *ex vivo* gene therapies has recently increased (Eggenhofer et al. 2012; Marofi et al. 2017; Iansante et al. 2021; Preda et al. 2021). One safety argument in favour of using hMSCs as an *ex* vivo gene therapy tool lies in fact that these cells are short lived when administered to humans (Eggenhofer et al. 2012; Muhammad et al. 2017). This makes gene-modified hMSCs a promising cellular modality in the scenario where a transient effect is desired. This strategy has been explored in studies targeting solid tumours or vascular disorders (Sage et al. 2014; Beegle et al. 2015, 2016; Yuan et al. 2016; Davies et al. 2019). Given the promising findings from pre-clinical studies using *ex vivo* gene-modified hMSCs, it is likely that some of these therapies will soon reach first-in-human studies.

The engineering challenge lies in reducing the cost of manufacturing. These products are overly expensive mostly due to the lack of robust and scalable manufacturing platforms. Manufacturing technologies which support commercial-scale demand are necessary to ensure the production of the final cellular therapeutic. The anchorage-dependent nature of hMSCs presents technical challenges as the cells need a surface to grow (Caplan 1991; Haynesworth et al. 1996). Therefore, their expansion in stirred tanks (STRs) depends on providing a suitable matrix (usually microcarriers) for cell adherence and suitable agitation levels. The use of microcarriers for hMSC culture in STRs has been proven extensively (Eibes et al. 2010; Santos et al. 2011; Rafiq et al. 2013; Tan et al. 2016; Rafiq et al. 2017b). Notably, none of these studies have investigated and demonstrated the production of gene-modified hMSCs.

Given the novelty of hMSCs-based *ex vivo *gene therapy applications, the challenge is to develop scalable manufacturing processes adapted to a product with these characteristics (Sage et al. 2014; Beegle et al. 2015, 2016; Yuan et al. 2016; Davies et al. 2019). The aim of this study was to investigate whether the expansion of transduced UCT-hMSCs can be performed using microcarriers and agitated conditions. The work involved the comparison between three groups: (1) non-transduced UCT-hMSC, (2) UCT-hMSC-GFP, and (3) UCT-hMSC-VEGF, evaluating the respective growth kinetics, metabolic profiles, and immunophenotype.

## Materials and methods

### Cell isolation

Fresh UCT samples were purchased from (Tissue Solutions, UK) and shipped at room temperature. An enzymatic digestion protocol was followed to perform the cell isolation work. Briefly, this involved exposing the perivascular tissue using a scalpel and surgical tweezers. The tissue was dissected into smaller pieces to facilitate the subsequent unit operations. The tissue was transferred to a shake flask (Corning, US), and an enzymatic solution (1 g/L) of collagenase NB4 (Serva, Germany) was added. The volumetric ratio between tissue and enzyme used was 1:1. The flasks were placed on a shaking platform inside an incubator (at 37 °C under 5% of CO_2_) and kept at 120 rpm for a maximum of three hours or until no tissue was observable. To neutralise the enzyme’s activity, expansion medium was added in a 1:1 ratio of enzyme to medium. Expansion medium was prepared by adding to Dulbecco’s modified Eagle’s medium (DMEM, 1 g/L glucose, Lonza, UK), 10% (v/v) foetal bovine serum (FBS) (Gibco, UK), and 2 mM UltraGlutamine (Lonza, UK). The mixture was centrifuged at 350 g for 5 min (Eppendorf, UK), after which the supernatant was removed, and the cell pellet was manually disturbed to eliminate cell clumping. Once the pellet was detached from the bottom of the conical tube, 100 mL of pre-warmed expansion medium was added. A final purification step was performed by filtering the cell suspension through a 60 μm steriflip centrifuge tube (Merck, Germany) to eliminate any undigested tissue left in suspension. A cell count was performed before the cells were either cryopreserved or expanded in monolayer.

### Cell expansion and cryopreservation

Immediately after isolation, two passages were performed to create a working cell bank of UCT-hMSCs. This was achieved by plating the cells at a density of 1 × 10^4^ cells/cm^2^ (P0-P1). A density of 5000 cell/cm^2^ was chosen for one subsequent passage (P2). The expansion was performed using the same culture medium formulation described above. When the cells reached 70–80% confluency, a new passage was initiated. The cryopreservation step was performed at the end of P2 after a cell detachment step using 0.25% (v/v) trypsin and 0.02% (w/v) EDTA solution (Gibco, UK) and followed by a centrifugation step using a 400 g cycle for 5 min. The cells were resuspended in CS10 (Biolife Solutions, USA) at the concentration of 1 × 10^6^ cells/mL.

### Plasmid purification

To produce the 2^nd^ generation LV, three different plasmids were used: pMD.G (envelope), P8.91 (packaging), and SIN pHV (GFP transgene) were previously described (Sanber et al. 2015) whereas the plasmids coding for VEGF (#89609; also encoding a GFP marker) were purchased from Addgene, USA. For the plasmids obtained from the Infection and Immunity Divison at UCL, a bacteria transformation step was performed using competent cells (DH5α, ThermoFisher, USA). For the plasmid purchased from Addgene, the bacteria stab was spread across Luria broth agar plates (Sigma, UK) followed by a 37 °C incubation overnight. The following morning, after confirming that the bacteria had grown, a single colony was picked and expanded first in Luria broth (Sigma, UK) until OD_600_ of 2.0 was reached. Then, the plasmids were isolated using a purification kit (QIAGEN, Germany) following the manufacturer’s protocol.

### LV manufacturing

Human embryonic kidney (HEK) 293 T cells were seeded at a density of 1 × 10^5^ cell/cm^2^ in DMEM with high glucose (Gibco, UK), 2 mM GlutaMAX (Gibco, UK) and 10% (v/v) FBS (Gibco, UK). One day after seeding, the packaging, envelope, and the plasmid carrying the transgene were mixed in a ratio of 1:1:1.5. In a separate reaction tube, 0.127 μL of FuGENE-6 (Promega, US) was mixed to 2.54 μL of Optimem (ThermoFisher, US) and then added to the cells. On the following day, a total medium exchange using the medium formulation described in this section. For the next three consecutive days, the vector-containing medium was harvested from the cell culture plate and filtered using a 0.45 μm polyethersulfone syringe filter (Merck, Ireland). The virus-containing medium was then stored at − 80 °C.

### Transduction of hMSCs

This unit operation started with thawing the UCT-hMSCs using a 37 °C water bath. The cells were cultured in monolayer for one passage, before the transduction was initiated (P4). The cells were then seeded at a concentration of 100,000 cell/cm^2^, and on the next day, the transduction step was performed using LV at multiplicity of infection (MOI, transduction units on HEK293T cells per a UCT-hMSC cell) of two using polybrene at a concentration of 8 µg/mL (Sigma, UK). The transduction efficiency was assessed using the methodology described in the “Analytical techniques” section.

### Expansion of hMSCs on microcarriers

For microcarrier-spinner flask culture, the microcarriers were prepared according to the manufacturer’s instructions. Preparation of the spinner flasks (BellCo Biotech, US) involved coating the vessels with Sigmacote® (Sigma, UK). Following the same experimental steps required during the microcarrier screening study, Spherecol microcarriers were prepared according to the manufacturer’s instructions. Briefly, the appropriate mass of Spherecol microcarrier was weighed to ensure 5 cm^2^ per mL of culture. The hydrated microcarriers were then autoclaved inside of the spinner flask. Before seeding, the expansion medium was used to remove residual water from the autoclave cycle (Richmond Scientific, UK). The UCT-hMSCs were then seeded in spinner flasks at a density of 6000 cells/cm^2^. To allow sufficient gas exchange whilst in the incubator, the side-arms of each spinner flask were loosened. Spinner flask agitation was provided by a Bell-Ennium Compact 5 (BellCo Biotech, USA). The initial cell-to-microcarrier attachment was performed with 50% of the working volume and with intermittent agitation: 25 min rest followed by 5 min at 30 rpm. This cycle was repeated for 8 h. One day after the initial seeding, expansion medium addition was performed to reach 100% working volume of the spinner flask. A medium exchange of 25% of the working volume was performed commencing from day three. This was performed by removing the spinner flask from the spinning platform allowing the microcarriers to settle to the bottom of the vessel by gravity. Then, the medium exchange was performed on the supernatant, without microcarriers or cells being removed.

### Analytical techniques

#### LV titre determination

To determine the concentration of the LVs produced, two methods were chosen: (1) a functional titre assay using flow cytometry and (2) physical titre assay that quantifies HIV-1 p24 antigen in cell culture supernatants via enzyme-linked immunosorbent assay (ELISA).

For the functional titre assay, HEK 293 T were plated at a concentration of 3.0 × 10^5^ cells per well using a 12-well plate. Polybrene was added to the cell suspension at a concentration of 8 μg/mL and the vector-containing medium in serial dilutions (1:10, 1:100, and 1:1000). One-day post-transduction, a medium addition was performed using 50% of the initial culture volume. Two days after, the cells were detached with 0.25% (v/v) trypsin and 0.02% (w/v) EDTA solution (Gibco, UK), centrifuged, and resuspended in Stain Buffer (BD Biosciences, UK). To quantify the functional LV concentration, GFP expression was then assessed using flow cytometry.

For the physical titre assay, an ELISA-based HIV-1 p24 antigen (Origene, US) assay was performed following the protocol established by the manufacturer. Briefly, the standard curve was prepared and the samples, went through incubation cycles with anti-HIV-1 p24 capture antibody, antibody detector, streptavidin HRP conjugate, and, finally, substrate. The final step was then absorbance measurement at 450 nm using a CLARIOstar plate reader (BMG Labtech, Germany). The analysis of the data relied on a standard curve that correlates absorbance to p24 concentration. This assay estimates that 1 pg of p24 equates to 1 × 10^4^ physical particles of LV.

#### Cell counts

Cell counting and viability were performed using a NucleoCounter NC-3000 (Chemometec, Denmark) Via1-Cassette™ (Chemometec, Denmark) were used. These cassettes have immobilised acridine orange (AO) and 4′,6-diamidino-2-phenylindole (DAPI) that enable the detection of cells and non-viable cells, respectively. AO is a permeable dye that binds directly to cell nuclei, whereas DAPI can only enter damaged cell membranes.

#### Cell viability assay

During the microcarrier screening stage, to overcome the limitations of automated cell counts when working at low concentrations, the WST-1 assay (Roche, Switzerland) was used. This colorimetric test uses the reduction of WST-1 by viable cells as an indirect measure for viable cell concentration determination. The WST-1 assay features a stable tetrazolium salt that is cleaved, leading to a soluble form of formazan, in a process occurring at the cell surface level. The amount of formazan dye in the supernatant can then be correlated to the metabolically active number of cells. Briefly, the WST-1 was added to the tissue culture well plates, containing cells attached to microcarriers, in a volumetric ratio of 1:10. The sample was kept for one hour in a humidified incubator at 37 °C and 5% CO_2_ and then analysed using a plate reader Infinite® 200 PRO (Tecan, Switzerland). The absorbance values were measured at 450 nm against a reference of 690 nm.

#### Metabolite analysis

The samples collected during the expansion stage were frozen at − 20 °C during the entire expansion cycle and thawed one hour before analysis. The CuBiAn® Bioanalyzer (Optocell, Germany) was used to determine the ammonia (mM), glucose (mM), and lactate (mM). The system was operated according to the manufacturer's instructions.

#### Transduction efficiency

Both lentiviral vector preparations carried a GFP marker gene, enabling transduction efficiency to be assessed for green fluorescence using flow cytometry. Briefly, the UCT-hMSCs were detached 72 h after LV exposure with 0.25% (v/v) trypsin and 0.02% (w/v) EDTA solution, centrifuged (400 g, 5 min) and resuspended in Stain Buffer (BD Biosciences, US). The percentage of GFP expressing cells was determined using a LSR Fortessa 1, (BD Biosciences, US). A non-transduced control was used to set the gate for the GFP expressing cells.

#### Immunophenotype characterisation

To perform a characterisation of the surface receptors on the expanded cells, a panel of markers based on ISCT criteria was followed. Briefly, a minimum of 1 × 10^5^ cells was used to perform a single staining protocol. This assay started with an incubation cycle using FC block (BD Biosciences, US), followed by another incubation step with the following antibodies: CD73-APC, CD90-APC, CD105-PE, CD11b-PE, CD19-APC, CD34-PE, CD45-PE-Cy5.5, and HLA-DR-PerCP-Cy7 all purchased from (BD Biosciences, US). The samples were then acquired using LSR Fortessa 1.

## Equations

Different equations were used to compare the growth kinetics and metabolism of the cells seeded in the different expansion platforms used in this study. All these equations were previously used in similar experimental setups across multiple studies (Rafiq et al. 2013; Dos Santos et al. 2014; Mizukami et al. 2016; Heathman et al. 2016)

1 $$\mu =\frac{Ln\left(\frac{cx\left(t\right)}{cx\left(0\right)}\right)}{\Delta t}$$

where *μ* represents the specific growth rate (d^−1^), $cx(t)$, and $cx(0)$ describe the total cell numbers at the end and the start of the exponential growth phase, respectively. Time was represented by $t (d)$.

2 $${t}_{d}=\frac{Ln(2)}{\mu }$$

where *t*_*d*_ represents doubling time (*d*) and *μ* represents the specific growth rate (d^−1^).

3 $$\mathrm{Fold increase} (FI)=\frac{cx(f)}{cx(0)}$$

where $cx(f)$ and $cx(0)$ correspond to final and initial cell concentration, respectively.

4 $${q}_{met}=\frac{\mu }{cx\left(0\right)}\times \frac{{C}_{met\left(t\right)}-{C}_{met\left(0\right)}}{{e}^{\mu t}-1}$$

where *q*_*met*_ represents specific metabolic consumption/production rate, *µ* specific growth rate (d^−1^), *C*_*met*(0)_ and *C*_*met*(*t*)_ correspond to the metabolite concentration at the start and end of the exponential growth phase, respectively, and $cx(0)$ is the cell number at the beginning of the exponential growth phase, whereas *t* represents time.

## Statistical analysis

To perform the statistical analysis required during the present work, the software SPSS (IBM, USA) was used. The Kruskal Wallis test was chosen to establish a comparison between the different groups studied. Significance levels were set at *P* values < 0.05 (*P-value < 0.05, **P-value < 0.01, ***P-value < 0.001, and ****P-value < 0.0001).

## Results

### Impact of different microcarriers on growth kinetics of UCT-hMSCs

**Fig. 1:**
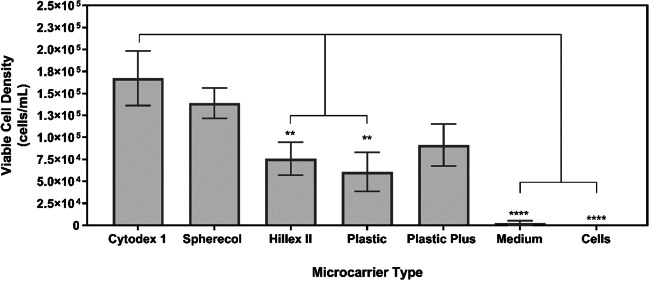
Cell concentration after the 8-day expansion of UCT-hMSCs. Data shown as mean with error bars representing standard deviation (*N* = 9). This study was performed using ultra-low attachment plates to avoid cell adhesion to the plates and enabling cell to microcarrier attachment

**Fig. 2:**
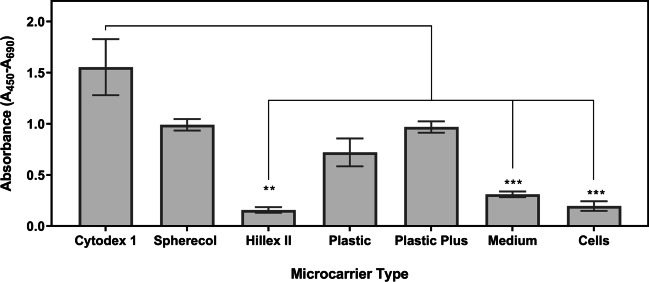
Absorbance levels resulting from the WST-1 assay after the 8-day expansion of UCT-hMSCs. Data shown as mean with error bars representing standard deviation (*N* = 9). This study was performed using ultra-low attachment plates to avoid cell adhesion to the plates and enabling cell to microcarrier attachment

**Fig. 3:**
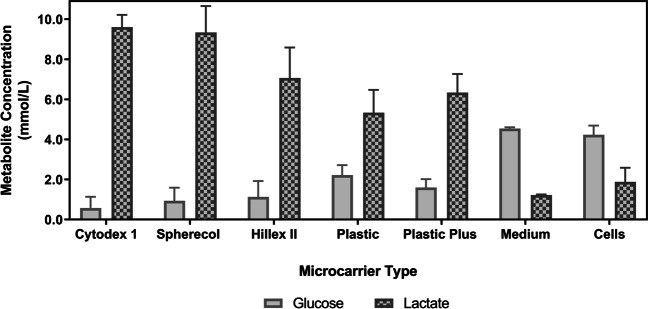
Glucose and lactate concentrations after an 8-day with UCT-hMSCs. Data shown as mean with error bars representing standard deviation (*N* = 9). This study was performed using ultra-low attachment plates to avoid cell adhesion to the plates and enabling cell to microcarrier attachment

Due to the adherent nature of hMSCs, a surface for cell adhesion needs to be provided to avoid cell death via anoikis (Vachon 2011). Given the focus of the present work on suspension culture, an initial screening experiment was conducted in monolayer and aimed to evaluate the performance of different microcarrier types on UCT-hMSC growth kinetics (Figs. 1, 2, and 3).

Figure 1 shows the cell concentration obtained after an eight-day expansion period using different microcarriers. Cytodex 1 and Spherecol were the microcarriers associated with the highest cell concentration during this study, followed by Hillex II, Plastic, and Plastic Plus. Given that several cell concentrations observed in the study are close to the lower limit of the optimal range of the equipment (5 × 10^4^ to 5 × 10^6^ cells/mL), an indirect cell quantification method was also performed (Fig. 2). The indirect cell counting assay also showed a similar trend, placing Cytodex 1 and Spherecol as the best performing microcarriers, followed by Plastic and Plastic Plus. It should be mentioned that Hillex II absorbed all the colours from the culture medium used. Therefore, this group was disregarded for the WST-1 assay interpretation purposes. This phenomenon was reported in a previous microcarrier screening study (Rafiq et al. 2016).

Metabolically active mammalian cells consume glucose and produce lactate during their expansion processes. The concentration of these metabolites was measured to gather additional information that can inform the selection of the microcarrier(s) that maximise cell concentration (Fig. 3). The metabolite analysis revealed that Cytodex 1 and Spherecol were associated with lower glucose concentrations after the eight-day expansion cycle. Conversely, the lactate concentration of these two groups was also the highest reported in this study. This suggests higher glucose consumption due to a more extensive proliferation of UCT-hMSCs. Taken together, these data sets suggest that Cytodex 1 and Spherecol maximise cell attachment and therefore potentiate improved growth kinetics when compared to the other microcarriers studied. Cytodex 1 is manufactured using a dextran matrix and uses diethylaminoethyl cellulose (DEAE) groups that are positively charged. Spherecol features polystyrene matrix coated with type I human collagen (Rafiq et al. 2016). Previous reports have highlighted that collagen supports cell adhesion to surfaces and proliferation of hMSCs (Schor and Court 1979; Heino 2007; Silva Couto et al. 2023). Given smaller variation across donors observed when using Spherecol, this microcarrier type was taken forward to perform the remaining suspension-based work.

### LV titration and transduction efficiency

Prior to transduction of the UCT-hMSCs, there was a LV manufacturing step performed. During this work, two LVs were prepared: one carrying a GFP marker only and another with a VEGF transgene and a GFP marker. Both these preparations were quantified using a physical and a functional method (p24 assay and infectivity assay, respectively). During the 3-day harvest period, a reduction in the functional titre was observed for both vector preparations, which is aligned with the trend reported in previous reports (Merten et al. 2016).

The average functional titre obtained was (5.91 ± 2.84) × 10^6^ TU/mL and (2.58 ± 1.24) × 10^6^ TU/mL for GFP and VEGF, respectively. The same decreasing trend during the 3-day harvesting was observed for the physical titre which was determined through quantification of p24. In this case, the average physical titre obtained was (2.12 ± 1.03) × 10^8^ VP/mL for (1.96 ± 0.72) × 10^8^ VP/mL for GFP and VEGF, respectively. Regarding the physical to functional particle ratios, these were 36.6 ± 5.62 and 80.7 ± 29.9 for the GFP- and VEGF-encoding LVs, respectively.

**Fig. 4:**
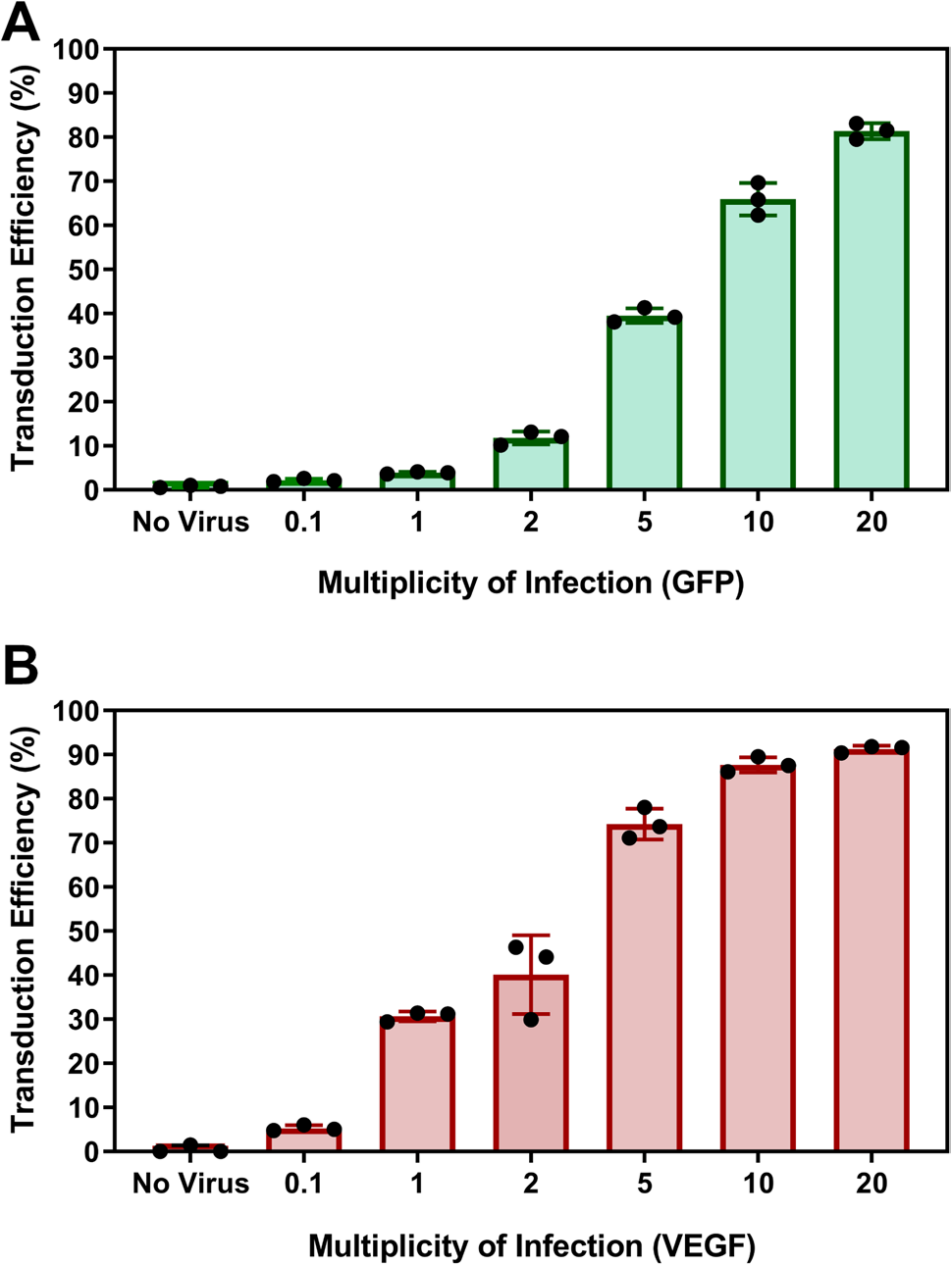
Transduction efficiency using different ratios of infections particles per cell for **A** GFP (top) and **B** VEGF (bottom). Data shows mean with error bars representing standard deviation (*N* = 3)

**Fig. 5:**
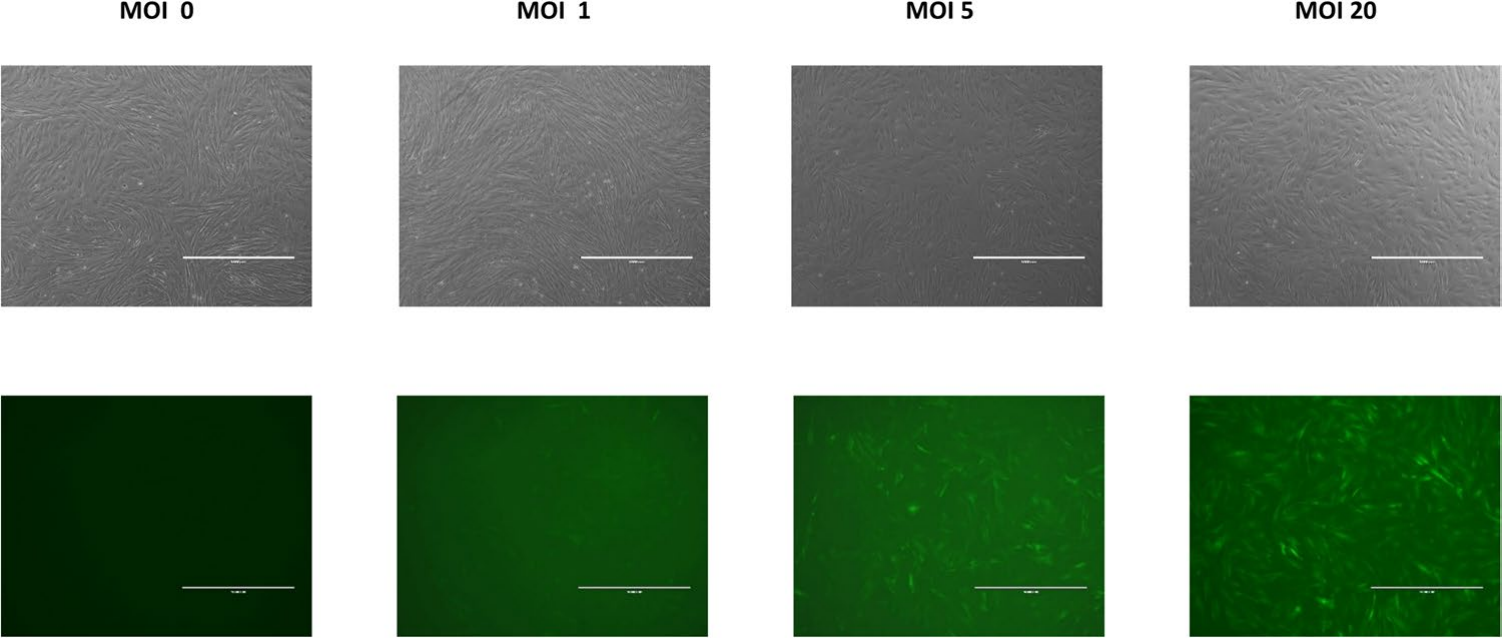
Phase contrast microscopy images of the UCT-hMSC-VEGF cells during the transduction efficiency study. MOIs of 0, 1, 5, and 20 represented in the figure (from left to right). Top line shows images collected with typical phase contrast microscopy settings applied and bottom line with a green filter turned on to allow GPF signal detection

To evaluate transduction efficiency when using these LV preparations, a titration study was conducted using MOI (LV 293 T transduction units to UCT-hMSCs ratios) between 0.1 and 20 (Figs. 4A, B and 5). When the percentage of transduced cells is below 40%, the number of integrations is approximate to the number of transduced cells. However, at higher MOIs, the number of transduced cells with multiple copy integrations increases, which may increase the risk for insertional mutagenesis. On this basis, an MOI of 2 was selected to provide high transduction efficiencies whilst minimising the risk of multiple integrations. Previous studies have demonstrated that increasing the MOI even just up to five led to an average of three insertions of the transgene per cell (Beegle et al. 2016).

### Growth kinetics of gene-modified UCT-hMSCs

To establish a scalable manufacturing process for gene-modified UCT-hMSCs, the transduced cells were expanded using microcarriers in suspension conditions in 80 mL spinner flasks. This study included a non-transduced control (UCT-hMSC) and two gene-modified cell populations, UCT-hMSC-GFP and UCT-hMSC-VEGF. Daily cell counts were performed throughout the 7-day expansion period to monitor cell growth kinetics.

**Fig. 6:**
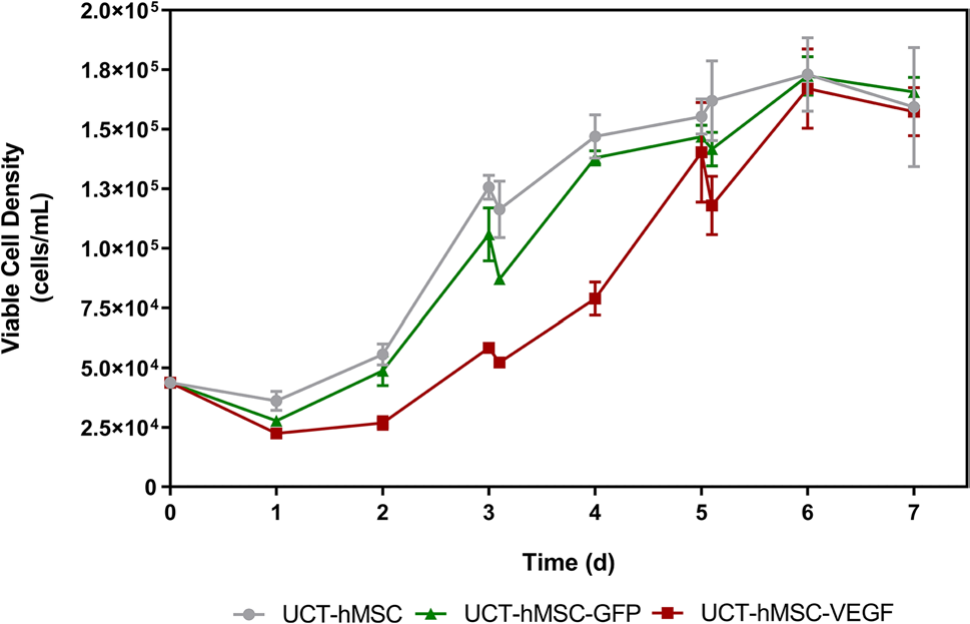
Viable cell concentration of non-transduced hMSCs and VEGF/GFP-transduced hMSCs expanded in microcarriers for 7 days. Data shows mean with error bars representing standard deviation (*N* = 3)

The expansion pattern of the three different cell populations revealed differences in the lag phase duration (Fig. 6). Whereas non-transduced UCT-hMSC and UCT-hMSC-GFP showed a lag phase of approximately one day, UCT-hMSC-VEGF showed a two-day lag phase. Some studies have optimised the manufacturing process of non-transduced hMSCs and reported cell growth starting from day one of culture (Dos Santos et al. 2014; Schirmaier et al. 2014; Mizukami et al. 2016; Tozetti et al. 2017; Rafiq et al. 2017a). However, a two-day lag phase was also reported previously (Eibes et al. 2010; Caruso et al. 2014; Yuan et al. 2014; Zhao et al. 2015; Chen et al. 2015; Lam et al. 2017; De Soure et al. 2017).

Despite the differences observed in the initial days of the expansion, all three cell groups studied (UCT-hMSC, UCT-hMSC-GFP, and UCT-hMSC-VEGF) achieved comparable maximum cell densities at similar time points (*N* = 3, *P*-value > 0.05). The maximum cell concentrations reached were 1.73 ± 0.15 × 10^5^ cells/mL, 1.72 ± 0.08 × 10^5^ cells/mL, and 1.67 ± 0.17 × 10^5^ cells/mL for UCT-hMSC, UCT-hMSC-GFP, and UCT-hMSC-VEGF, respectively. These cell yields were comparable to previously reported studies conducted at the 100 mL scale (Sun et al. 2010; Carmelo et al. 2015; Li et al. 2015; Mizukami et al. 2016). Although the non-transduced control and UCT-hMSC-GFP displayed similar growth rates (0.281 ± 0.131 d^−1^ and 0.317 ± 0.126 d^−1^, respectively), the UCT-hMSC-VEGF group was demonstrated to grow at a faster rate 0.457 ± 0.045 d^−1^ (*N* = 3, *P*-value < 0.05). These three groups have shown growth rates comparable to some studies previously published (Eibes et al. 2010; Santos et al. 2011; Yuan et al. 2014). The doubling time for UCT-hMSC, UCT-hMSC-GFP and UCT-hMSC-VEGF was also calculated at 2.44 ± 0.305 d, 2.19 ± 0.181 d, and 1.52 ± 0.151 d, respectively. These results suggest that the expansion of gene-modified hMSCs has been successfully performed, reaching similar cell concentrations published in previous studies.

### Metabolic profile of gene-modified UCT-hMSCs

**Fig. 7:**
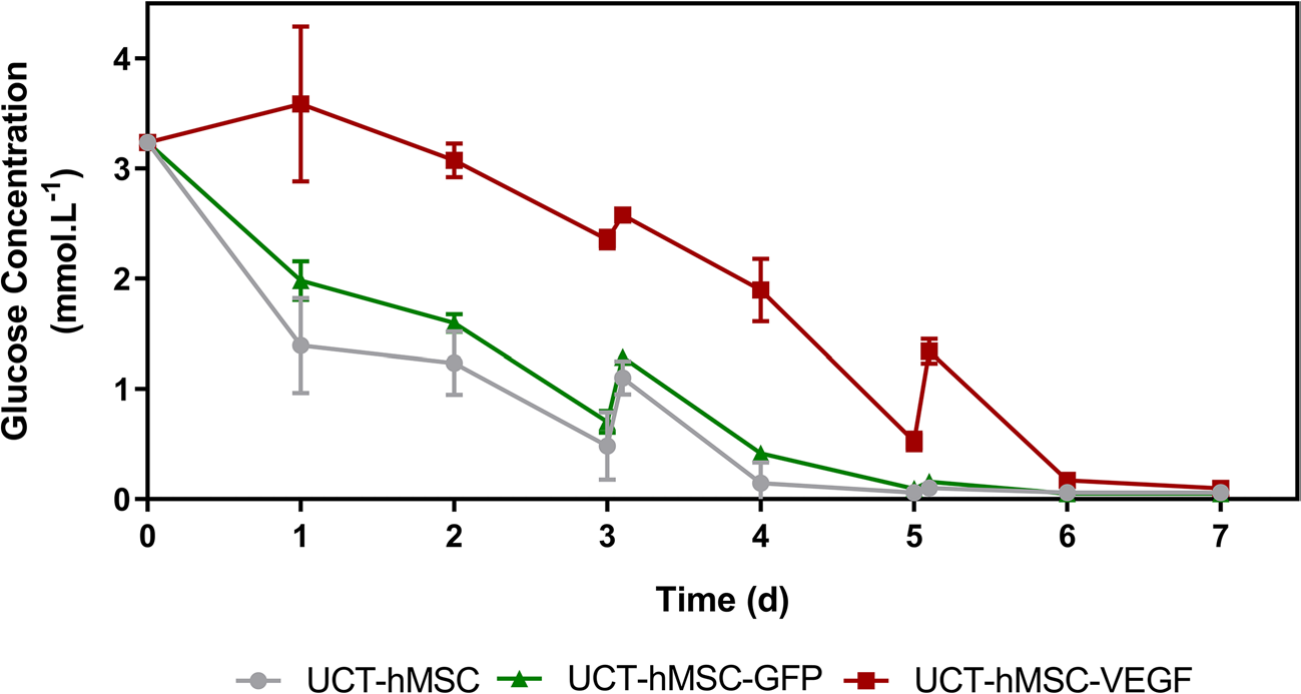
Glucose concentration profile of non-transduced hMSCs and VEGF/GFP-transduced hMSCs expanded in microcarriers for 7 days. Data shows mean with error bars representing standard deviation (*N* = 3)

Daily measurements of glucose, lactate, and ammonia were performed to evaluate the metabolic consumption/production patterns during the expansion phase. Firstly, it was observed that glucose concentration (Fig. 7) decreased gradually during cell expansion in all three groups studied (UCT-hMSC, UCT-hMSC-GFP, and UCT-hMSC-VEGF). It was also noticed that both UCT-hMSCs and the GFP-transduced cells experienced glucose levels close to 0 mM by day 4. These results are aligned with the viable cell density, where the growth rate for UCT-hMSC and UCT-hMSC-GFP reduces from day four onwards. This is potentially attributable to the lack of glucose availability in the medium. In contrast, UCT-hMSC-VEGF only reached similar glucose levels on day 6. These results are indicative that glucose availability may be one of the limiting factors of cell growth in the three groups studied. The average consumption rate was calculated during the exponential growth phase to understand the glucose consumption pattern on a cell basis. No difference was reported in the glucose consumption rates obtained across non-transduced (77.98 ± 52.93 pmol·cell^−1^·day^−1^) and transduced cell populations (UCT-hMSC-GFP: 77.98 ± 52.93 pmol·cell^−1^·day^−1^, UCT-hMSC-VEGF: 213 ± 134 pmol·cell^−1^·day^−1^) (*N* = 3, *P*-value > 0.05).

**Fig. 8:**
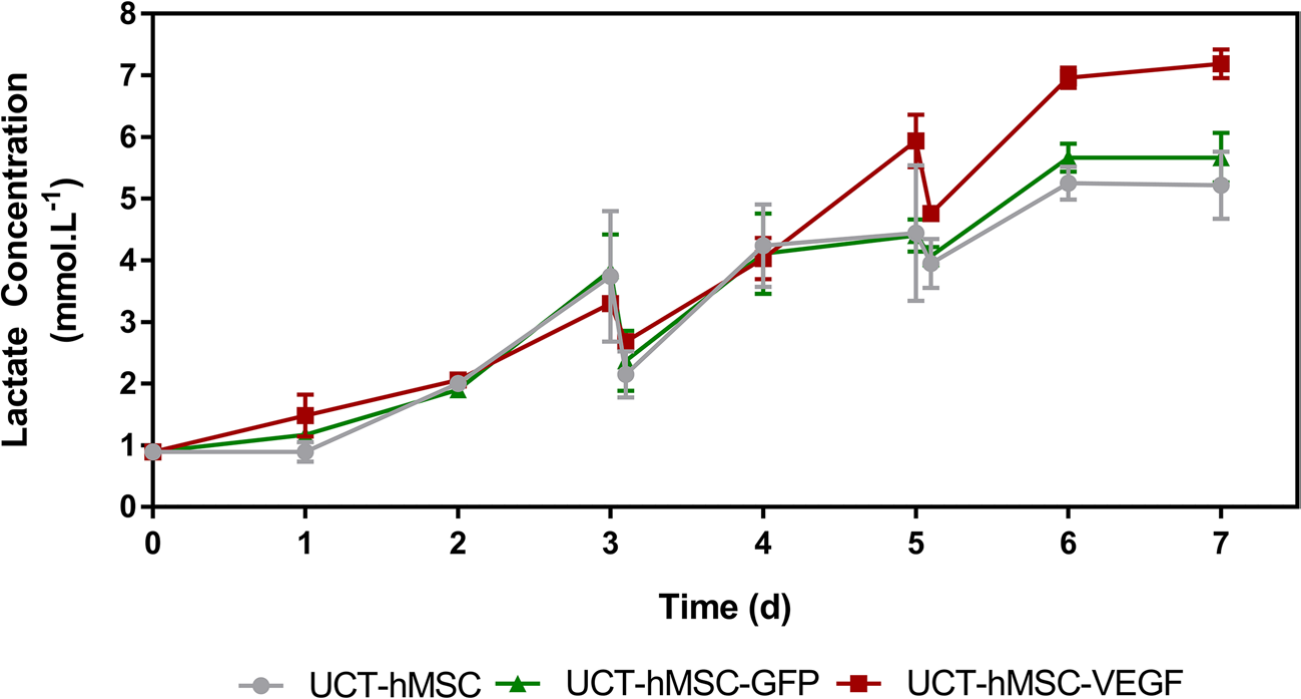
Lactate concentration profile of non-transduced hMSCs and VEGF/GFP-transduced hMSCs expanded in microcarriers for 7 days. Data shows mean with error bars representing standard deviation (*N* = 3)

Given that lactate is formed during the glycolysis via glucose degradation (Pattappa et al. 2011; Barilani et al. 2019), its concentration was measured daily throughout the culture (Fig. 8). At the end of the 7-day expansion period, both the non-transduced and the UCT-hMSC-GFP reached lactate concentrations of 5.21 ± 0.54 mM and 5.67 ± 0.41 mM, respectively. In this same period, a higher lactate concentration was reported in the UCT-hMSC-VEGF group (7.19 ± 0.23 mM) (*N* = 3, *P*-value < 0.05). These concentrations are aligned with those reported in other studies (Rafiq et al. 2013; Dos Santos et al. 2014; Carmelo et al. 2015). Noteworthy, these values are considerably lower than the levels reported to inhibit hMSCs growth (Schop et al. 2010). The average consumption rate was calculated during the exponential growth phase to understand the lactate production pattern per cell basis. Comparable glucose consumption rates were obtained across non-transduced (126 ± 103 pmol·cell^−1^·day^−1^) and transduced cell populations (GFP-164 ± 141 pmol·cell^−1^·day^−1^, VEGF-361 ± 141 pmol·cell^−1^·day^−1^) (*N* = 3, *P*-value > 0.05). Lactate concentration did not have any significant changes from day six onwards as the medium formulation had no glucose left to be consumed.

**Fig. 9:**
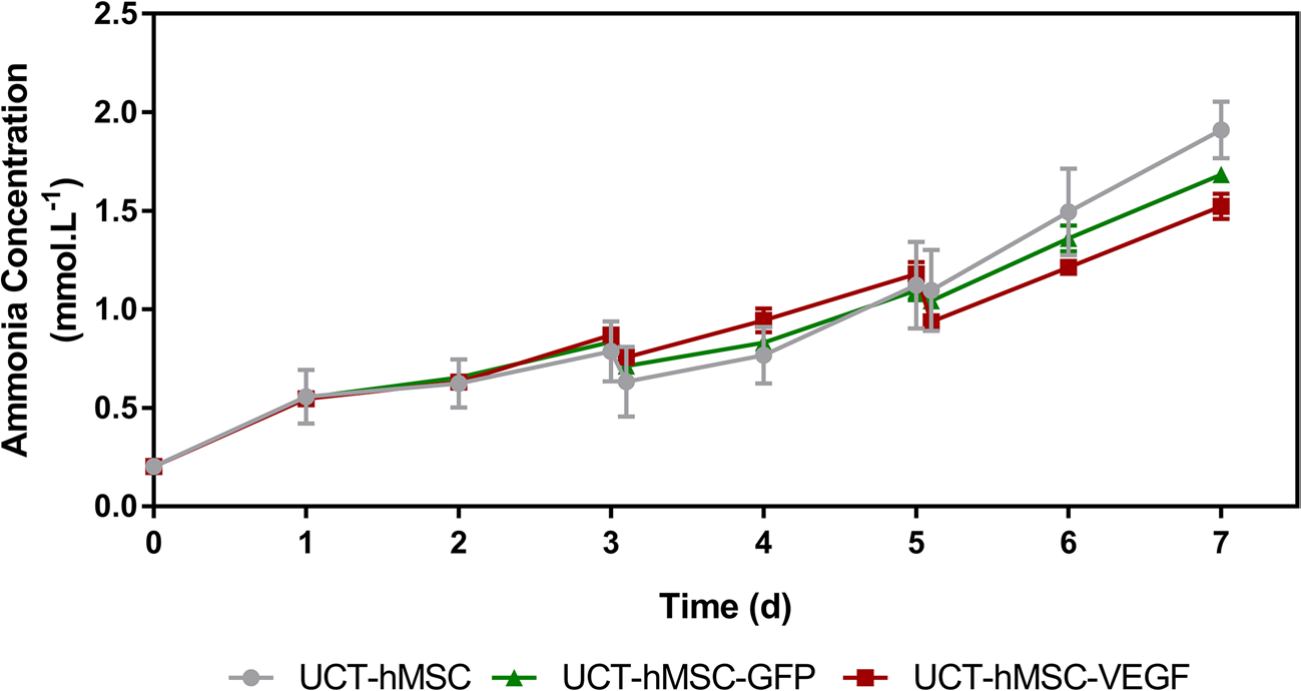
Ammonia concentration profile of non-transduced hMSCs and VEGF/GFP-transduced hMSCs expanded in microcarriers for 7 days. Data shows mean with error bars representing standard deviation (*N* = 3)

A similar analysis was conducted for ammonia, a common by-product usually traced due to its key role during amino acid breakdown (Genzel et al. 2008; Salazar et al. 2016) (Fig. 9). The concentration of this metabolite increased over the 7-day culture period with UCT-hMSC (1.91 ± 0.14 mM) reaching the highest concentration reported, followed by UCT-hMSC-GFP (1.68 ± 0.03 mM) and finally UCT-hMSC-VEGF (1.52 ± 0.06 mM). It is important to note that the maximum ammonia concentration obtained in this study was below the threshold reported to inhibit hMSC growth (Schop et al. 2010). This suggests that ammonia accumulation is unlikely to have caused the cells to reach stationary phase. Regarding the production rate per cell basis, all groups studied showed comparable ammonia consumption patterns with 4.61 ± 1.79 pmol·cell^−1^·day^−1^, 4.28 ± 3.19 pmol·cell^−1^·day^−1^, and 4.13 ± 0.85 pmol·cell^−1^·day^−1^ for UCT-hMSC, UCT-hMSC-GFP, and UCT-hMSC-VEGF, respectively (*N* = 3, *P*-value > 0.5).

### Immunophenotype characterisation

**Fig. 10:**
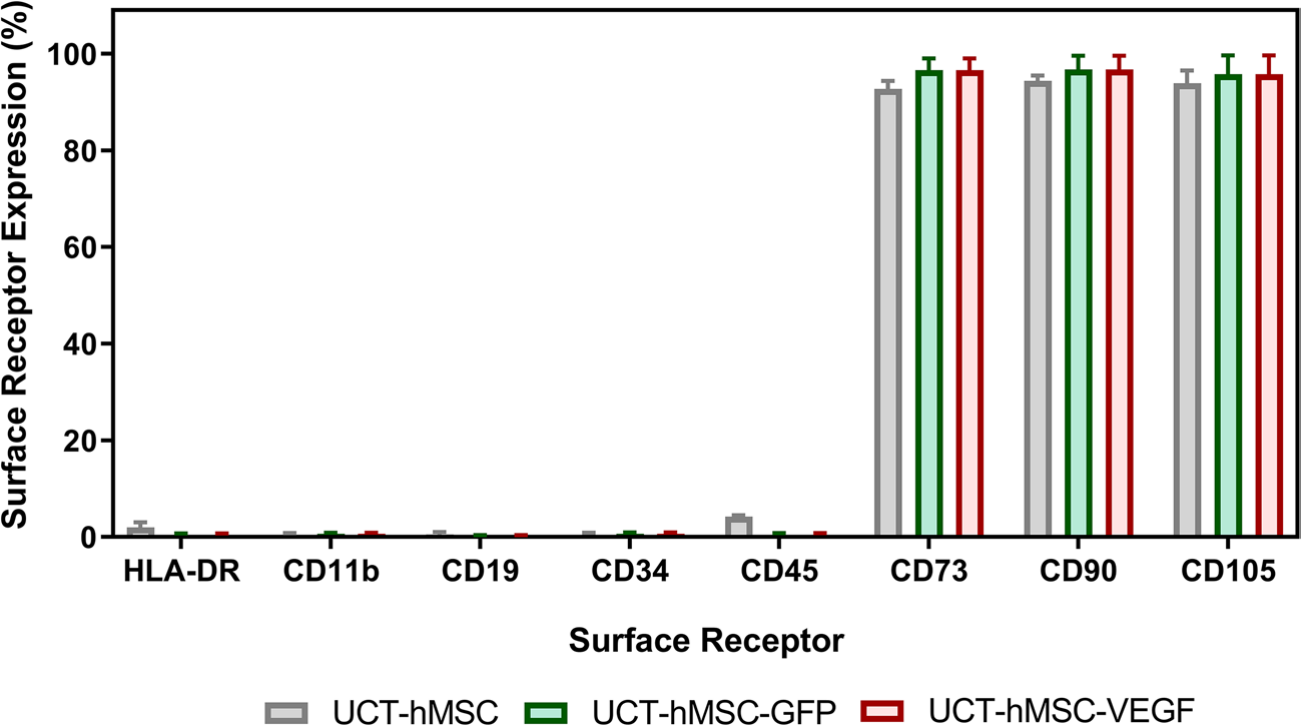
Immunophenotype characterisation of non-transduced and GFP/VEGF transduced hMSCs after expansion in microcarriers for 7 days. Data shows mean with error bars representing standard deviation (*N* = 3)

To evaluate the immunophenotype of both non-transduced and gene-modified hMSCs, flow cytometric analysis was performed on termination of the expansion cycle (Fig. 10). Both transduced (UCT-hMSC-GFP and UCT-hMSC-VEGF) and non-transduced cells shown more than 90% of expression levels for the markers CD73, CD90, and CD105. On the other hand, CD11b, CD19, CD34, CD45, and HLA-DR expression levels was below 5%. Given the role of CD73 in conversion of adenosine monophosphate (AMP) into adenosine (Yang et al. 2020) and CD90 critical role in cell–matrix and cell–cell adhesion (Kisselbach et al. 2009), it is unsurprising that the expression of this receptor remained unaltered post transduction/expansion. Similarly, CD105 have been reported to play a critical role in angiogenesis (Duff et al. 2003). It is therefore reasonable to assume that this manufacturing process did not lead to a downregulation of this receptor either. This indicates that the integration of the transgene did not lead to any insertional mutagenesis capable of down- or upregulate genes responsible for hMSCs’ immunophenotype (Vranckx et al. 2016; Milone and O’Doherty 2018).

### Comparison with monolayer

To assess how the suspension-based system and a monolayer process would compare, growth kinetics and metabolite analysis was performed across expansion systems for all three groups studied (UCT-hMSC, UCT-hMSC-GFP, and UCT-hMSC-VEGF) (Table 1). When expanded in monolayer, no difference was found between non-transduced and transduced cells. Although UCT-hMSC-VEGF showed a faster proliferation ability across the cell populations studied when expanded in microcarrier, this same trend was not observed in monolayer. It can be hypothesised that UCT-hMSC-VEGF may have similar growth kinetics to the other groups studied. Nevertheless, during the expansion in a spinner flask may have faced additional challenges during the attachment stage. This was observed in Fig. 5 where non-transduced cells and UCT-hMSC-GFP showed a lag phase of one day, whereas UCT-hMSC-VEGF took two days before cell growth was noticeable. The fold expansion in the monolayer culture was considerably higher than in the one obtained in the spinner flasks. However, given that spinner flasks were operated with 80 mL of working volume, the total number of cells produced is approximately 13 million *versus* close to 1 million in the monolayer (using a T25 flask). This represents a strong argument favouring developing manufacturing processes in suspension culture that can be easily scalable. Conversely, monolayer culture is generally reliant on scale-out to increase its production capacity.

**Table 1:** Comparison table of the different metrics assessed during the expansion of non-transduced and gene-modified hMSCs both in a spinner flask and in a monolayer

Cellular Product	Process parameters	Static monolayer	Agitated spinner flask
UCT-hMSCs	Maximum cell concentration (× 10^5^/mL)	–	1.73 ± 0.15
	Days to reach maximum density (d)	–	6
	Total cell yield (× 10^6^ cells)	1.09 ± 0.05	13.8 ± 0.1
	Doubling time (d)	2.24 ± 0.18	2.44 ± 0.30
	Fold expansion	8.72 ± 0.41	3.95 ± 0.35
	Specific growth rate (d^−1^)	0.309 ± 0.05	0.281 ± 0.131
	Glucose consumption rate (pmol·cell^−1^·d^−1^)	1911 ± 53	77.98 ± 52.93
	Lactate production rate (pmol·cell^−1^·d^−1^)	4187 ± 777	126 ± 103
	Yield of lactate from glucose (mol.mol^−1^)	2.19 ± 0.36	2.07 ± 0.53
	Ammonia production rate (pmol·cell^−1^·d^−1^)	982 ± 152	4.61 ± 1.79
	Minimum glucose level (mM)	0.14 ± 0.12	0.061 ± 0.01
	Maximum lactate level (mM)	9.49 ± 1.51	5.21 ± 0.55
	Maximum ammonia level (mM)	1.41 ± 0.14	1.91 ± 0.14
UCT-hMSC-GFP	Maximum cell concentration (× 10^5^/mL)	–	1.72 ± 0.08
	Days to reach maximum density (d)	–	6
	Total cell yield (× 10^6^ cells)	0.98 ± 0.01	13.7 ± 0.12
	Doubling time (d)	2.35 ± 0.15	2.19 ± 0.18
	Fold expansion	7.87 ± 0.86	3.94 ± 0.18
	Specific growth rate (d^−1^)	0.295 ± 0.08	0.317 ± 0.126
	Glucose consumption rate (pmol·cell^−1^·d^−1^)	1997 ± 50	87.49 ± 5.98
	Lactate production rate (pmol·cell^−1^·d^−1^)	4727 ± 336	164 ± 141
	Yield of lactate from glucose (mol·mol^−1^)	2.36 ± 0.15	2.37 ± 1.97
	Ammonia production rate (pmol·cell^−1^·d^−1^)	964 ± 7.14	4.28 ± 3.17
	Minimum glucose level (mM)	0.23 ± 0.02	0.071 ± 0.01
	Maximum lactate level (mM)	9.68 ± 1.03	5.66 ± 0.40
	Maximum ammonia level (mM)	1.84 ± 0.09	1.68 ± 0.03
UCT-hMSC-VEGF	Maximum cell concentration (× 10^5^/mL)	–	1.67 ± 0.17
	Days to reach maximum density (d)	–	6
	Total cell yield (× 10^6^ cells)	1.02 ± 0.01	13.6 ± 0.12
	Doubling time (d)	2.31 ± 0.37	1.52 ± 0.15
	Fold expansion	8.12 ± 0.88	3.81 ± 0.38
	Specific growth rate (d^−1^)	0.299 ± 0.01	0.457 ± 0.041
	Glucose consumption rate (pmol·cell^−1^·d^−1^)	1901 ± 26	213 ± 134
	Lactate production rate (pmol·cell^−1^·d^−1^)	4030 ± 67	361 ± 1410
	Yield of lactate from glucose (mol·mol^−1^)	2.11 ± 0.04	1.78 ± 0.91
	Ammonia production rate (pmol·cell^−1^·d^−1^)	838 ± 59	4.13 ± 0.85
	Minimum glucose level (mM)	0.36 ± 0.07	0.091 ± 0.01
	Maximum lactate level (mM)	7.05 ± 0.10	7.19 ± 0.24
	Maximum ammonia level (mM)	1.42 ± 0.29	1.52 ± 0.06

Culture duration in the monolayer groups was five days, which corresponded to 80% confluency. A seven-day expansion period was established for the spinner flask-based cultures given that the maximum cell concentration was observed on day 6

Although glucose concentration was close to being fully depleted, it was marginally higher than in the agitated condition. This trend was observed both in transduced and non-transduced cells. It was also observed that the lactate concentration at the end of the culture period was higher in static conditions than in suspension-culture for UCT-hMSCs, UCT-hMSC-GFP, and UCT-hMSC-VEGF. The ammonia concentration in the cell culture supernatants from static cultures was comparable to the suspension systems. Notably, neither lactate nor glucose reached inhibitory concentration as defined previously (Schop et al. 2009). The lactate yield from glucose from each group was comparable between suspension and static conditions. This suggests that, irrespective of the expansion platform and the cells’ genetic modification status, they were still likely to be following the glycolysis pathway.

## Discussion

In this manuscript, microcarriers and spinner flasks were used to expand gene-modified hMSCs carrying process development and clinically relevant transgenes. This set of studies demonstrated that LVs can be successfully used to transduce hMSCs and those can be expanded in suspension culture.

In this study, Cytodex and Spherecol microcarriers enabled cell growth to superior levels compared to the other microcarriers studied (Hillex II, Plastic, and Plastic II). Previous research focused on evaluating the impact of different microcarriers in hMSC growth kinetics have shown plastic, fibronectin, and collagen coated microcarriers as the best performers (Goh et al. 2013; Kaiser et al. 2013; Carmelo et al. 2015; Petry et al. 2016; Rafiq et al. 2016). It can be hypothesised that these observations are related to the critical role that bioactive molecules such as fibronectin and collagen have cell-to-matrix adhesion processes. These have previously been associated with enhanced cell proliferation (Singh and Schwarzbauer 2012; Somaiah et al. 2015; Salzig et al. 2016; Maerz et al. 2016; Smeriglio et al. 2017; Basoli et al. 2021)**.**

LV manufacturing represents a significant bottleneck of based *ex vivo* gene therapies. As such, this work also evaluated the ratio between functional and physical particles of both LV preparations generated. The ratios obtained in this work (~ 37 and 81 for GFP and VEGF-containing vectors, respectively) are aligned with previously reported ratios for LV production using second-generation LV systems (McCarron et al. 2019). The ratio of infections to physical particles typically insight into the quality of the vector preparation, with a lower ratio being more desirable due to the lower quantity of non-infectious particles (McCarron et al. 2019).

After transduction, the growth kinetics of gene-modified hMSCs seeded in spinner flasks was evaluated. Although non-transduced and GFP-transduced hMSCs showed comparable growth kinetics, a longer lag phase was reported in the UCT-hMSC-VEGF group. This observation may be attributable to inefficient attachment of cells to microcarriers as previously described (Tsai et al. 2020). To maximise cell attachment to microcarriers, parameters, such as working volume used during the attachment stage or agitation mode, were previously investigated (Yuan et al. 2014; Takahashi et al. 2017; Rafiq et al. 2017a). In addition, previous studies demonstrated that donor variability might also play a critical role in cell growth kinetics and the duration of their lag phase (Hupfeld et al. 2014; Santhagunam et al. 2014).

It should be noted that bioprocess development studies previously conducted have reported higher cell concentrations than those obtained in this work (Sun et al. 2010; Chen et al. 2015; Rafiq et al. 2017a). One of the potential explanations for this disparity may be due to key process variables, in particular, donor-to-donor variability. Other variables that can explain these differences include culture medium, tissue source, microcarrier type, attachment mode, agitation rate, feeding, and control strategy (Silva Couto et al. 2020b; Delbridge et al. 2023a, b). Additionally, this is the first study demonstrating the feasibility of using spinner flasks and microcarriers to expand gene-modified hMSCs whilst the wider literature has focused on primary hMSC expansion.

Although a comparable maximum cell density was observed between the three groups studied, the UCT-hMSC-VEGF were shown to grow faster than the non-transduced cells and UCT-hMSC-GFP. Given that this difference was not observed in the monolayer control flasks, it was hypothesised that this observation was related to the longer lag phase registered in the UCT-hMSC-VEGF group. Despite the differences observed in growth kinetics, glucose consumption as well as ammonia and lactate production were comparable between the three cell preparations studied.

Daily measurements of glucose, lactate, and ammonia were performed to evaluate these metabolites’ consumption/production patterns. UCT-hMSC-VEGF showed a different metabolic profile than the other two groups. As such, two options can help explain these observations: (1) the differences observed are related to the nature of the genetic modification introduced; and (2) the longer lag phase observed in the UCT-hMSC-VEGF group impacts the growth rate and consequently the metabolic consumption patterns. It is difficult to ascertain whether the differences shown at a metabolic level are directly related to the genetic modification or to the lag phase. It was previously described that LV integration is not completely random and each class of retrovirus has its preferential insertion location (Lewinski et al. 2006; Ciuffi 2016; Milone and O’Doherty 2018). Therefore, it seems unlikely that the genetic modification is the root cause of the metabolic difference noticed between UCT-hMSC-VEGF and the remaining groups. In addition, given that the MOI in this study was kept as low as two, it is unlikely that multiple copies of the transgenes (GFP or VEGF) have been inserted (Wahlers et al. 2001). This strategy was previously adopted to minimise the risk of generating genetically unstable cell populations due to multiple insertions of the transgene. Altogether, this suggests that one of the reasons for the different growth kinetics observed between UCT-hMSC, UCT-hMSC-GFP, and UCT-hMSC-VEGF was the more extended lag phase observed in the latter. The rationale behind this observation remains unclear, but the hypothesis that this is due to other factors unrelated to the genetic modification should not be excluded. To identify what was the root cause for the difference noted in growth kinetics, additional studies using different LV preparations could also be performed. This would help to identify whether there is any relation to the gene introduced.

Whilst glucose concentration was higher, lactate and ammonia levels herein reported were lower than previous publications, possibly due to the slower growth rates reported in this work. It should be mentioned that the baseline levels of glucose in the medium formulation used in this work were lower than reported elsewhere (Eibes et al. 2010; Rafiq et al. 2013; Mizukami et al. 2016; Rafiq et al. 2016; Lam et al. 2017). In all of the groups (UCT-hMSC, UCT-hMSC-GFP and UCT-hMSC-VEGF), glucose depletion was not observed, and lactate and ammonia concentrations were maintained below the inhibitory threshold. The yield of lactate from glucose observed across the three studied groups was close to 2 mol/mol both in agitated and static conditions. Future optimisation studies focused on the feeding strategy need to be performed to reach cell yields closer to some of the latest studies reported in the literature.

It was also observed that neither the transduction nor the expansion step led to changes in the cellular immunophenotype. Therefore, CD73, CD90, and CD105 expression levels were above 90% for UCT-hMSC, UCT-hMSC-GFP, and UCT-hMSC-VEGF. In addition, the expression of negative markers (CD11b, CD19, CD34, CD45, and HLA-DR) was also shown to be below 5% which is aligned with the criteria established for hMSCs by the ISCT (Dominici et al. 2006). It was hypothesised that glucose was the limiting factor that prevented further cell growth. This was supported by the glucose concentration profile that reached values close to zero on day 5 for UCT-hMSC and UCT-hMSC-GFP and day 6 for UCT-hMSC-VEGF. Both lactate and ammonia did not reach inhibitory concentrations according to previously published studies (Schop et al. 2009). In this work, a decrease in the percentage of GFP positive and VEGF positive hMSCs was observed (supplementary material). A few causes were hypothesised to explain this observation: (1) different attachment properties between transduced and non-transduced cells to microcarriers, (2) distinct growth kinetic profiles between transduced and non-transduced cells, and (3) transient expression of the GFP and VEGF transgenes. Although this study demonstrated that spinner flasks and the microcarrier approach enabled the expansion of gene edited hMSCs, a limitation of this work is the absence of a functional assay for the UCT-hMSC-VEGF *ex vivo* gene therapy candidate, i.e., quantification of VEGF concentration in the cell culture supernatant.

The immunophenotype was comparable across experimental conditions, and these match the expression levels defined by ISCT for hMSCs (Dominici et al. 2006). This demonstrates that introducing a genetic modification using a second-generation LV system followed by a microcarrier-based expansion step, did not change cellular immunophenotype. Similar findings were previously reported in a study that included a wider array of surface receptors (Al-Nbaheen et al. 2013). In this work, the authors demonstrated that after transduction with a LV carrying the human telomerase reverse transcriptase gene (hTERT), the immunophenotype of the cells generated was similar to primary hMSCs isolated from different tissues. Additional efforts conducted to assess the impact of gene-editing on hMSCs’ characteristics revealed that the differentiation potential might be upregulated after insertion of the transgene (Hung et al. 2004; Tsai et al. 2010). From a safety perspective, it is key to ensure that the final product is non-tumorigenic and non-immunogenic. Although it was outside the scope of this work, it was previously demonstrated that VEGF-transduced hMSCs did not exhibit tumorigenic properties nor led to chromosomal aberrations even when operating at an MOI of 20 (Beegle et al. 2015, 2016). It is key to highlight that whilst working with UCT-hMSC-VEGF the expected MoA relies on the action of the transgene inserted and its ability to initiate angiogenesis (Beegle et al. 2016). In another study focused on evaluating the ability of hMSCs as ex vivo gene therapy for solid tumours, it was demonstrated that although the non-transduced cells have limited pro-apoptotic effect, TRAIL-transduced hMSCs were capable of inducing apoptosis of tumour cells (Loebinger et al. 2009; Lathrop et al. 2015; Guiho et al. 2016). Both studies reinforce the evidence that the key transgenes introduced constitute the backbone of *ex vivo* gene therapies. In this work, a tri-lineage differentiation assay was not conducted, given its lack of relevance in ascertaining the potency of a UCT-hMSC-VEGF product.

This study demonstrates, for the first time, the feasibility of expanding gene-modified hMSCs in a scalable microcarrier-bioreactor manufacturing platform. Initially, it was demonstrated that second-generation LV systems manufactured using a packaging plasmid (P8.91), an envelope plasmid (pMD2.G), and a plasmid carrying the transgenes GFP and VEGF were capable of infecting UCT-hMSCs at MOIs ranging from 0.1 to 20. It was further demonstrated that both UCT-hMSC-GFP and UCT-hMSC-VEGF reached similar concentrations to the ones obtained for non-transduced UCT-hMSC after a 7-day expansion period. Moreover, in suspension conditions, all groups studied reached the maximum cell density at the same time point (day 6) exhibiting comparable immunophenotypic characteristics.

## Supplementary Information

Below is the link to the electronic supplementary material.

Supplementary file1 (PDF 196 KB)

## Data Availability

All data is made available and presented in the manuscript.
