# Supplementary material for: Scalable manufacturing of gene-modified human mesenchymal stromal cells with microcarriers in spinner flasks
Source: Appl Microbiol Biotechnol. 2023 Jul 20;107(18):5669–85. doi: 10.1007/s00253-023-12634-w (PMC10439856; doi:10.1007/s00253-023-12634-w)
Supplement: Supplementary file 1 — Supplementary file1 (PDF 196 KB) [file 253_2023_12634_MOESM1_ESM.pdf]

Applied Microbiology and Biotechnology

Scalable manufacturing of gene-modified human mesenchymal stromal cells with microcarriers in spinner flasks

Pedro Silva Couto[1], Dale J. Stibbs[1], Marco C. Rotondi[1], Yasuhiro Takeuchi[2,3], Qasim A. Rafiq[1*]

[1]Department of Biochemical Engineering, Advanced Centre for Biochemical Engineering, University College London, Gower Street, WC1E 6BT, London, United Kingdom

[2]Division of Infection and Immunity, University College London, Gower Street, WC1E 6BT, London, United Kingdom

[3]Biotherapeutics and Advanced Therapies, Scientific Research and Innovation, Medicines, and Healthcare products Regulatory Agency, EN6 3QG, South Mimms, UK

*Corresponding Author

*Qasim Rafiq: q.rafiq@ucl.ac.uk +44 (0) 203 108 4420 (telephone), +44 (0) 20 7679 9603 (fax)

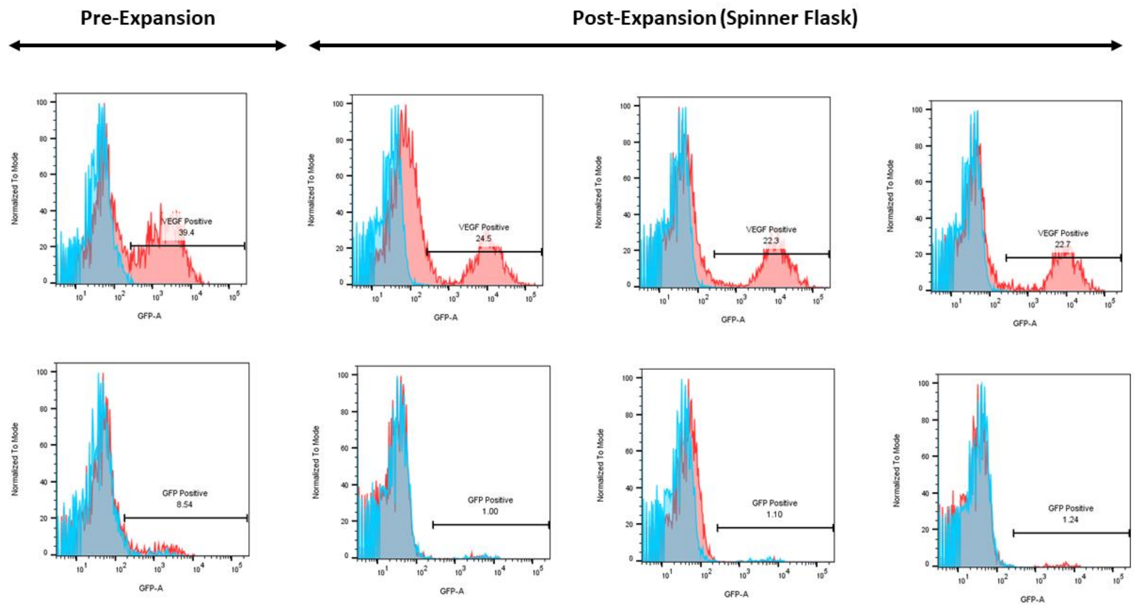

Figure S1-Histograms of GFP positive cells for UCT-hMSC-VEGF (top) and UCT-hMSC-GFP (bottom).
